# The Widening Fields of Public Health

**Published:** 1921-06-11

**Authors:** C. E. A. Winslow

**Affiliations:** Director, Department of Health, League of Red Cross Societies, and Professor of Public Health, Yale University.


### THE WIDENING FIELDS OF PUBLIC HEALTH.

By C. E. A. WINSLOW, Director, Department of Health, League of Red Cross Societies, and Professor of Public Health, Yale University.

An extensive system of new social machinery has been evolved during the last decade to meet the pressing demand for health education. Lectures, exhibits, cinemas, brochures, newspaper propaganda, have been created to produce a general atmosphere favourable to the movement. For the most essential task of personal instruction to the individual in the home, the new professions of public health nurse and health visitor have been created, and have met with such success that, particularly in America, the public health nurse, who adds to her fundamentally necessary training in the care of the sick a special training or experience in the teaching of hygiene, has come to be perhaps the central figure in the public health campaign. Above all, through the organisation of anti-tuberculosis leagues, and associations for the prevention of infant mortality and social hygiene societies and the like, and still more recently by the development of the peace-time health programme of the Red Cross, a vast body of laymen and laywomen has been brought into active and organic union with the public health movement as a whole.

### Prevention and Cure.

One of the things which makes life most interesting is the fact that the solution of one problem usually presents us with several new problems to be answered. In the field of public health the success of the educational campaign for personal hygiene brought forward in diverse forms the whole question of the relation between the preventive and curative medicine. In the past, prevention and cure had often been kept wholly distinct. Prevention was the affair of the State, treatment (except for the pauper) a private matter. The teacher of hygiene soon found, however, that instruction, in order to be effective, must be definite and personal. Food, fresh air, exercise, and rest are indeed the four pillars of the Temple of Health; but the question of real importance is the quantitative application in each individual case. What is important for A-B is to know how much and what kind of food, how much and what kind of exercise, how much and what kind of rest, he needs in order to keep his particular bodily machine in the best working order. Such instruction as this should, obviously, be based on a medical diagnosis. The health teacher needs the physician, if the work is to be well done.

### Opportunity for the Red Cross.

Among physicians themselves, there are equally strong forces working for the closest liaison with the public health campaign. Only through a system of organised clinical service can medical resources be utilised to the best advantage for the prevention rather than the amelioration of disease. It is a recognition of these facts that has led to the remarkable growth of child-welfare clinics, school clinics, nutrition classes, mental clinics, tuberculosis clinics, venereal clinics, and the like, during the past few years.

In this connection, again, the lay health organisation, and particularly the Red Cross Society, can render an invaluable service to the cause of public health. Clinics and hospitals alike will ultimately form part of an official system of State medicine. The inception of new enterprises of this kind is, however, very properly the task of volunteer organisations, and there is no greater opportunity for any national Red Cross, in the way of peace-time service, than the initiation and demonstration of the value of these essential first-aid stations in the war against disease.

### The Vicious Circle.

Poverty and disease operate in a vicious circle—sickness as a cause of destitution, destitution as a factor in sickness. The great sanitary awakening in Great Britain owed its original impetus to Chadwick's belief that the control of preventable disease would go far to reduce the burden of poor relief. It is equally true, however, that while considerable groups in the population attempt to subsist on an income below the minimum essential for physical well-being, the relief of economic stress will form an essential part of the public health programme. In dealing with the problems of infant mortality, of tuberculosis, or of venereal disease, the constructive efforts of the social worker must sooner or later be called into play.

Public health is the science and the art of preventing disease, prolonging life, and promoting physical health and efficiency through organised community efforts for the sanitation of the environment, the control of community infections, the education of the individual in principles of personal hygiene, the organisation of medical and nursing service for the early diagnosis and preventive treatment of disease, and the development of the social machinery which will ensure to every individual in the community a standard of living adequate for the maintenance of health.

### Crusade of All Peoples.

The programme here suggested is no light one, and yet there is no single element in it which can be neglected if we are to conserve the precious resources of human vitality. If such a programme is to be realised, even in moderate measures, it must require not only the services of experts—not only the support of States—but the whole-hearted co-operation of the men and women who make up those States and whom those experts serve.

To enlist that co-operation in the age-long struggle against disease and death is the great mission to which the Red Cross Societies are pledging their mighty energies. This is in very truth the new crusade to which the peoples of the earth are called.

